# The natural history of recent hepatitis C virus infection among blood donors and injection drug users in the country of Georgia

**DOI:** 10.1186/s12985-016-0478-6

**Published:** 2016-02-03

**Authors:** Tengiz Tsertsvadze, Lali Sharvadze, Nikoloz Chkhartishvili, Lela Dzigua, Marine Karchava, Lana Gatserelia, Akaki Abutidze, Kenrad E. Nelson

**Affiliations:** Faculty of Medicine, Ivane Javakhishvili Tbilisi State University, 16 Al. Kazbegi Avenue, Tbilisi, 0160 Georgia; Infectious Diseases, AIDS and Clinical Immunology Research Center, 16 Al. Kazbegi Avenue, Tbilisi, 0160 Georgia; Georgian-French Joint Hepatology Clinic ‘Hepa’, 16 Al. Kazbegi Avenue, Tbilisi, 0160 Georgia; Bloomberg School of Public Health, Johns Hopkins University, 615 N. Wolfe Street, Room E7638, Baltimore, Maryland 21205 USA

**Keywords:** HCV, Natural history, Seroconversion, Transitory

## Abstract

**Introduction:**

Hepatitis C virus (HCV) infection is a serious health problem in Georgia.

**Methods:**

We conducted a prospective study to identify and characterize the natural history of recent HCV infection since very first days of infection. Recent HCV infection was defined as detectable plasma HCV RNA in the absence of anti-HCV antibodies.

**Results:**

A total of 7600 HCV seronegative blood donors and 3600 HCV seronegative drug users were screened for recent HCV infection. Among them 7 (0.09 %) blood donors and 10 (0.28 %) drug users tested positive for HCV RNA and were classified as having recent HCV infection. Of these 17 patients 4 (23.5 %) spontaneously cleared the virus by the end of 24 week follow-up. Five clinical forms of recent HCV infection were identified during the follow-up. Four patients had symptomatic disease, including 3 patients with jaundice and other clinical symptoms (2 of them cleared virus) and 1 patient only had other symptoms without jaundice. All symptomatic patients had ALT elevation. Three distinct variants of asymptomatic disease were identified in 13 patients: 9 patients had ALT elevation and none cleared the virus; 2 patients developed chronic disease without ALT elevation; 2 patients cleared virus without anti-HCV seroconversion and without ALT elevation; this form can be described as transitory HCV viremia.

**Conclusion:**

Additional studies are needed to define clinical and public health implications of transitory HCV viremia. Our study suggests the need for implementing nucleic acid testing of blood donors and key populations in order to more effectively identify HCV infected persons.

## Background

An estimated 185 million people are living with hepatitis C virus (HCV) globally and up to 500,000 deaths are attributed to the disease annually [1, 2]. Approximately 25 % of cases HCV infections are self-limited resulting in spontaneous clearance after acute infection, with the remaining 75 % of patients progressing to chronic disease [3]. Up to one third of patients with chronic hepatitis C develop liver cirrhosis and/or hepatocellular carcinoma (HCC) [3–5].

Although there is no protective vaccine against HCV, current treatment modalities offer high cure rates, which are expected to further improve with more powerful therapies currently in development [6]. This has brought promise that HCV can be eliminated or more effectively prevented through treating patients with hepatitis C, a so called treatment as prevention strategy [7]. However, there are serious challenges, including access to HCV testing, care and treatment services. For example, in the United States only half of the estimated 3.5 million people living with HCV infection were aware of their status and only 16 % of them were prescribed treatment [8]. The access is even more limited in resource-poor countries [9]. In addition, the risk of re-infection after successful curative therapy, which is substantial among injection drug users (IDU), should also be taken into account [10].

Despite the tremendous progress in the field of hepatitis C, knowledge about the natural course of acute disease since the very early days of infection remains limited. Acute HCV is frequently asymptomatic making it very difficult to identify patients early in the course of disease. Therefore, studying acute HCV infection, prior to their seroconversion, by detecting seronegative subjects who are HCV RNA seropositive is an important method of early detection and can provide additional insights into the immunology and virology of acute phase of the infection.. From a public health standpoint, identification of factors associated with recent infection can help to better understand drivers of transmission in order to design preventive strategies.. Altogether improved knowledge of recent HCV infection can contribute to the development of interventions that can prevent establishment of chronic infection.

Georgia is an independent country located in Eastern European region between Russia and Turkey. Georgia experienced a rapid increase in the IDU population in the 1990s, which has resulted in a high HCV prevalence in the country [11]. Based on population-based survey an estimated 6.7 % of the adult population of the capital city of Tbilisi has hepatitis C, with prevalence reaching 70 % among IDUs [11, 12]. The other group found to be at risk of HCV in Georgia were paid blood donors, [13] who have been identified to be at high risk of blood borne infections in other settings as well [14].

The objective of the present study was to identify and characterize the course of recent HCV infection by identifying and prospectively studying patients who were HCV RNA positive prior to anti-HCV seroconversion.

## Methods

We conducted a 3-year prospective observational study to identify and follow patients with recent HCV infection, defined as detectable plasma HCV RNA in the absence of anti-HCV antibodies. For this purpose we tested anti-HCV seronegative blood donors and anti-HCV seronegative IDUs for qualitative detection of HCV RNA in plasma. Blood donors were initially screened for HCV at blood banks, while IDUs received their first HCV test at HIV testing and counseling sites.

We applied a mini-pool approach to screen for HCV RNA by testing a pool of 6 samples. Non-reactive pools were excluded from further investigation, while reactive pools were further resolved by testing each individual sample in the pool. Remaining individual blood specimens from initial screening were used for mini-pool testing.

Detection of HCV RNA in anti-HCV negative persons gave us the unique possibility to identify early HCV infection in the seronegative window period (before seroconversion) and to follow the natural course of disease.

All HCV RNA positive persons underwent additional investigations, including plasma HCV RNA quantification (viral load), HCV genotyping, ALT level determination and clinicalimitatl examination immediately after identification their recent HCV infection. Patients were followed for 6 months and anti-HCV, viral load, ALT, bilirubin and clinical/physical examinations were repeated at 2, 4, 8, 12 and 24 weeks of follow-up.

Because of the risk of co-infections with HIV or HBV all patients with recent HCV infection were tested for infection with these viruses. However, none of them had either infection.

Spontaneous HCV clearance was defined as two consecutive negative HCV RNA results after a confirmed recent HCV infection. The time point when spontaneous viral clearance occurred was defined as the midpoint between the date of the last detectable measurement of HCV RNA and the date of the first of two consecutive samples with undetectable HCV RNA. The time point when anti-HCV seroconversion occurred was defined as the midpoint between the date of the last negative and the first positive anti-HCV test. ALT elevation was defined as a level of >40 U/l,

All patients with recent infection were interviewed to collect demographic and epidemiological information. The probable mode of HCV transmission was assigned based on the reported risk factors by applying the presumed hierarchical order of probability of acquiring the infection. Percutaneous exposure, including IDU, was considered as the strongest risk factor for acquisition. Sexual transmission was assumed if a person reported no risk behavior other than having sexual intercourse with person known to be HCV positive. The mode of transmission was classified as undetermined if a person reported no risk factors for acquiring HCV.

Screening for HCV antibodies was conducted using the ORTHO HCV 3.0 enzyme linked immunosorbent assay (EIA). Qualitative detection of HCV RNA was performed using COBAS AMPLICOR HCV Test, v2.0 (Roche Diagnostics, Germany) which has a lower detection limit of <50 IU/ml; viral load was measured using COBAS TaqMan HCV 2.0 Test (Roche Diagnostics, Germany) which has a lower detection limit of <10 IU/ml; HCV genotype was determined using reverse hybridization line probe assay (INNO LiPA) using VERSANT HCV Genotype kit 2.0 (Innogenetics, Belgium). All laboratory assays were performed in accordance with the manufacturer’s instructions.

The study was approved by the institutional review boards (IRB) of the Infectious Diseases, AIDS and Clinical Immunology Research Center and Johns Hopkins University. Informed written consent was obtained from all persons.

## Results

A total of 7600 HCV seronegative blood donors and 3600 HCV seronegative IDUs were screened for recent HCV infection. Among them 7 (0.09 %) blood donors and 10 (0.28 %) IDUs tested positive for HCV RNA and were classified as having recent HCV infection.

Baseline characteristics of 17 patients with recent HCV infection are summarized in Table 1. Briefly, the majority was men (88.2 %) and their median age was 31 years. Twelve patients (including 2 blood donors) were infected through IDU (70.6 %), one patient acquired HCV via sexual contact (5.9 %) and the mode of transmission could not be determined in four patients (23.5 %). Overall, 59 % of patients were infected with an HCV genotype 1 virus. Overall, 4 (23.5 %) patients spontaneously cleared the virus by the end of the 24 week follow-up.

**Table 1:** Baseline characteristics of patients with recent HCV infection (*n* = 17)

	Total Population (*n* = 17)	Patients with spontaneous clearance (*n* = 4)	Patients with chronic infections (*n* = 13)
Age, median years (range)	31 (22–39)	30 (22–31)	32 (24–39)
Sex, *n* (%)			
Men	15	3 (20.0)	12 (80.0)
Women	2	1 (50.0)	1 (50.0)
Probable mode of HCV transmission, *n* (%)			
Injection drug use	12	2 (16.7)	10 (83.3)
Sexual contact	1	1 (100.0)	0 (0.0)
Undetermined	4	1 (25.0)	3 (75.0)
HCV genotype, *n* (%)			
Genotype 1a	1		1 (100.0)
Genotype 1b	9	2 (22.2)	7 (77.8)
Genotype 2a/2c	3	1 (33.3)	2 (66.7)
Genotype 3a	4	1 (25.0)	3 (75.0)

**Fig. 1:**
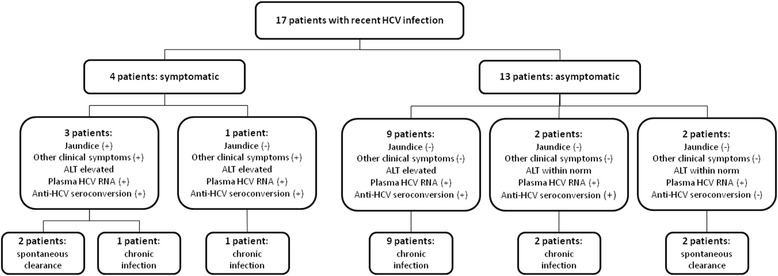
Clinical and laboratory variants of recent HCV infection (*n* = 17)

Five clinical forms of recent HCV infection were identified from the follow-up studies (Fig. 1).

Two variants of symptomatic disease were identified (Fig. 1). In 3 patients the disease was clinically manifested by jaundice and other clinical symptoms; while 1 patient only had other clinical symptoms without jaundice. These symptoms included fatigue, malaise, nausea, fever and arthralgia. Symptoms occurred after 4–8 weeks of follow-up. All 4 symptomatic patients had elevated levels of ALT, with median peak value of 1218 IU/l (range: 410–2050). Bilirubin was elevated in all 4 patients, ranging between 3.6 and 8.0 mg/dl at peak levels in patients with jaundice, and reaching 1.8 mg/dl in patient with other clinical symptoms only. All of these subjects experienced anti-HCV seroconversion after a median of 6 weeks (range: 3–10). Of these 4 patients, 2 spontaneously cleared the virus. One patient, carrying genotype 1b HCV, cleared the virus by week 12. In another patient with genotype 2a/2c virus plasma HCV RNA became negative at week 24.

Three distinct clinical and laboratory variants of asymptomatic disease were identified in 13 patients (Fig. 1). The first group consisted of 9 patients with elevated levels of ALT (the peak median ALT value was 957 IU/l [range: 220–1685]). Three patients had slightly elevated levels of bilirubin not exceeding 2 mg/dl. All of these 9 patients experienced anti-HCV seroconversion and developed chronic disease.

Two asymptomatic patients seroconverted and developed chronic disease without elevation of ALT or bilirubin over the follow-up period (Fig. 1). So, these two patients developed chronic HCV infection without any clinical or laboratory evidence of hepatitis during the observation period. The median time to seroconversion among 11 asymptomatic patients was 6 weeks (range: 3–18).

Two asymptomatic patients cleared the virus without anti-HCV seroconversion and without ALT elevation (Fig. 1). One of these patients cleared the virus by week 2 (genotype 1b) and another by week 4 (genotype 3a). Both patients remained negative for plasma HCV RNA and anti-HCV at the week 24 evaluation. Thus these two cases can be described as transitory HCV viremia.

**Fig. 2:**
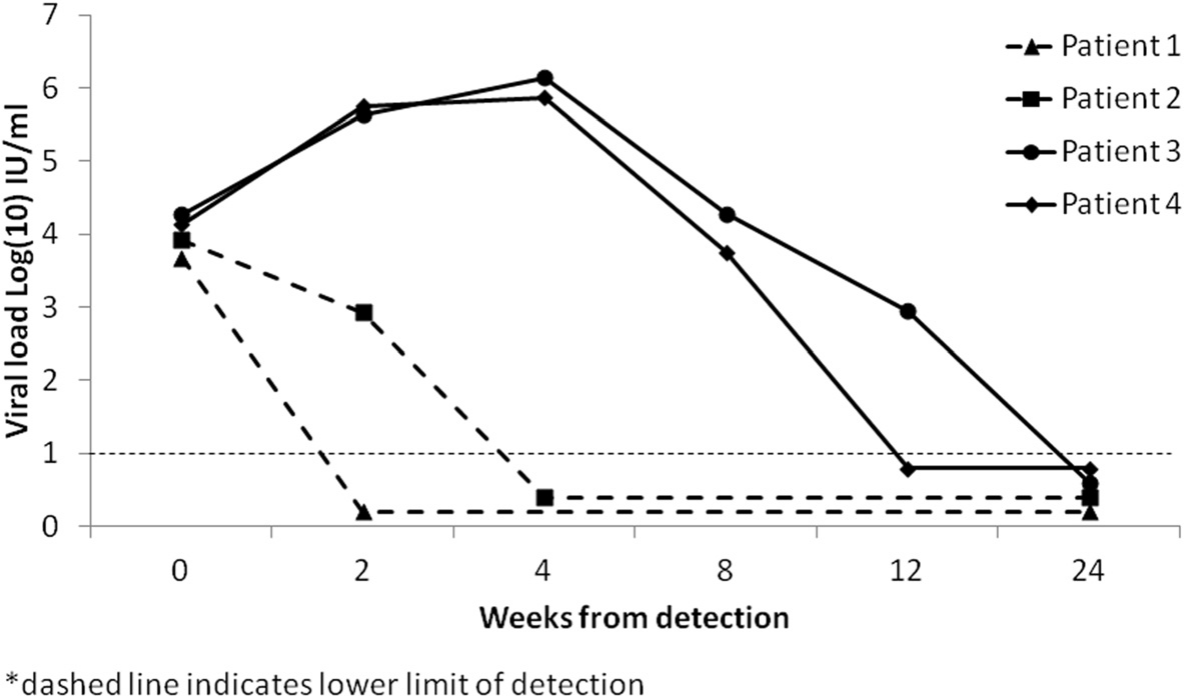
HCV viral load dynamics among patients with spontaneous viral clearance (*n* = 4)

**Fig. 3:**
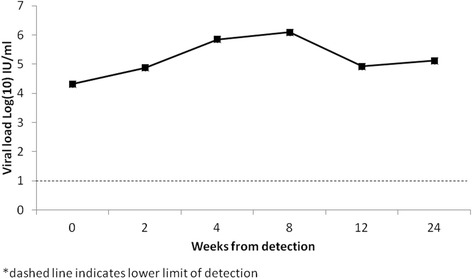
HCV viral load dynamics among patients with chronic infection (average value, *n* = 13)

Analysis of HCV viral load dynamics showed that patients with transitory viremia had lower initial levels of viral load – 3.68 log_10_ IU/ml in patient 1, who cleared the virus by week 2, and 3.92 log_10_ IU/ml in patient 2 who cleared the virus by week 4 (Fig. 2). Among the other two patients who cleared the virus, the initial viral load was 4.27 log_10_ IU/ml in patient 3 and 4.14 log_10_ IU/ml in patient 4 (Fig. 2). Patient 3 and 4 achieved peak viral load at week 4 with 6.14 log_10_ IU/ml and 5.87 log_10_ IU/ml respectively (Fig. 2). Patients with persistent infection had a slower pace of increase in the viral load, with peak median value registered at week 8–6.10 log_10_ IU/ml (Fig. 3). A total of 9 patients with chronic infection had viral load values of at least 6 log_10_ IU/ml over the follow-up.

## Discussion

We report the results of a unique study that followed patients with recent HCV infection after the early days of infection. We were able to identify these cases among seronegative persons using qualitative PCR, which was further confirmed by quantification of HCV RNA in plasma. Our study described two very unusual trajectories of acute HCV infection which may be more common than have been appreciated in clinical studies.

The first is the case of transitory viremia, when 2 patients, positive for HCV RNA, spontaneously cleared the virus without developing any clinical symptoms, without ALT elevation and most surprisingly without anti-HCV seroconversion. To the best of our knowledge there have been only a few reports of a similar abortive HCV infection [15–18]. In our study, one patient remained viremic for up to 2 weeks of follow-up, and another patient—for up to 4 weeks of follow-up. The duration of viremia in all of the above mentioned studies was short, and therefore identification of these cases was possible only through special design applied in studies. It is not surprising that cases of transitory HCV viremia have not been frequently identified since the majority of studies only use recent anti-HCV seroconversion and ALT elevation as key criteria for defining acute HCV infection [19]. Given the above mentioned difficulties related to identification of transitory viremia, it remains unknown how frequent transitory viremia and what are its clinical and public health implications or whether such patients have any immunity to subsequent HCV.

The other interesting form of acute HCV identified in our study was the two asymptomatic patients with recent infection who progressed to chronic hepatitis C without elevation of ALT. Both patients experienced seroconversion within the 24 weeks of follow-up. ALT elevation, as an evidence of clinical hepatitis, occurs shortly after the increase in the plasma HCV RNA levels [3]. Although published data indicates that persistently normal ALT during chronic HCV infection is not rare, [20, 21] elevation of ALT is normally expected in established infection regardless of the initial clinical presentation. Stramer and colleagues described three cases of long-term HCV viremia for up to 3 year period without elevated ALT, but these patients did not seroconvert during the follow-up [15].

With regard to other clinical forms of disease, 4 patients had symptomatic disease, including 3 patients who became jaundiced, and two of them spontaneously cleared the virus. Symptomatic disease, especially the occurrence of jaundice, has been shown to be associated with higher rates of spontaneous clearance of HCV [22, 23]. There were 9 asymptomatic patients with elevated levels of ALT and anti-HCV seroconversion, none of whom cleared the virus. This is not entirely surprising, given that asymptomatic disease without elevated biochemical markers of hepatitis has been associated with the lowest rate of spontaneous clearance, i.e about 10 % [23].

Overall the spontaneous viral clearance rate in our study was 23.5 %, which is similar to the rate of 26 % estimated in a large systematic review [22]. Higher rates of viral clearance of more than 40 % were reported more recently from Brazil [24] and Egypt [25]. However, substantial proportion of the population of these cohorts was symptomatic woman. Both symptomatic disease and female sex have been associated with a more favorable outcome of acute HCV infection, [22], including higher clearance rates.

We found similar patterns of viral load dynamics of the subjects in our study as other studies have reported [3, 26–28]. Patients with spontaneous clearance, excluding cases of transitory viremia, and those with persistent infection had similar peak levels of viral load, but the slope of increase in viral load early in the course of infection was higher among patients who cleared the infection. This result supports a recent finding by Liu and colleagues [29] indicating an association between high initial viral load and spontaneous clearance. However, we should be cautious with this conclusion, since in our study there were only four cases of viral clearance, of which two patients had transitory HCV viremia.

Our study identified another very important public health challenge—the safety of blood and blood products when donors have only been screened serologically. The national program on blood safety in Georgia was implemented in 1997 and currently ensures screening of all donated blood for HIV, HBV, HCV and syphilis. Implementation of this program resulted in a significant reduction of transfusion transmitted infections (TTI). However, as seen in our study the program failed to detect 7 blood donors with recent HCV due to the fact that the national program relies only on antibody detection against this infection. Since the late 1990s nucleic acid testing (NAT) of donated blood become a gold standard in high income countries, which minimized the residual risks of transmission of viral agents [30]. Our study provides evidence that justifies the introduction of NAT testing of blood donors in Georgia. This is particularly important for HCV taking into consideration its high prevalence in the country and longer seronegatve window period than HIV [11].

In addition to NAT testing donor selection remains a key strategy for achieving the safety of the blood supply. The majority of blood donors in Georgia are commercial donors, who are at higher risk of HCV and other TTIs [13]. In our study, of 7 blood donors with recent HCV infection 2 were IDUs. During the study interview they disclosed a recent history of sharing of injecting paraphernalia, but they did not report this fact before donating blood. Efforts are needed to increase voluntary donations and implement effective screening algorithm that accurately identifies blood donors with high risk behaviors.

Our study has several limitations. First, a small number of patients with recent HCV infection were identified, which substantially limited the statistical power of the study to assess associations and make meaningful comparisons. The second possible limitation is the relatively short duration of follow-up. It is well established that the vast majority of patients with spontaneous clearance after acute HCV infection clear the virus within 6 months of infection, [3, 4] subsequently we limited the follow-up in our study to 24 weeks. However, there are reports showing viral clearance after 6 and even 12 months periods even in patients with established chronic infection [31–33]. Thus it is possible that we missed some patients who might have cleared the virus after the end of follow-up. Our study was not powered to evaluate immunological aspects of recent HCV infection, which might have provided explanations for rare clinical cases seen in this study. Finally, it is now clear that interleukin 28B (IL28B) plays an important role in spontaneous clearance of acute HCV, [34] but unfortunately stored samples have not been available to test for IL28B retrospectively.

## Conclusions

In summary, our study identified and followed cases of recent HCV infection after very early in their infection. We described two of acute HCV, namely abortive HCV infections without further seroconversion and a subject with persistent infection without ALT elevation. Additional studies are needed to define the clinical and public health implications of transitory HCV viremia. Our study emphasizes the urgent need for improving the safety of the blood supply by implementing NAT testing and promoting voluntary donations.

## Abbreviations

HCV: Hepatitis C virus
HCC: Hepatocellular carcinoma
IDU: Injection drug users
EIA: Enzyme linked immunosorbent assay
IRB: Institutional review boards
TTI: Transfusion transmitted infections
NAT: Nucleic acid testing
IL28B: Interleukin 28B
